# Parent-adolescent closeness predicts neurophysiological reward responsiveness in adolescent girls at varying risk for depression

**DOI:** 10.1016/j.dcn.2025.101579

**Published:** 2025-06-03

**Authors:** Julianne M. Griffith, Anna Wears, Nastasia O. McDonald, Jennifer S. Silk, Rebecca B. Price, Mary L. Woody

**Affiliations:** a University of Pittsburgh School of Medicine, Department of Psychiatry, USA; b University of Pittsburgh, Department of Social Work, USA; c University of Pittsburgh, Department of Public Health, USA; d University of Pittsburgh, Department of Psychology, USA

**Keywords:** Reward positivity, Event-related potentials, Adolescence, Depression, Parent-adolescent relationships

## Abstract

Risk for depression rises during adolescence, particularly among children of depressed mothers. Altered neurophysiological reward processing, measured using event-related potentials (ERPs), is related to depression vulnerability. However, it is unclear whether disruptions in youth reward responsiveness are driven by parental reward dysfunction (e.g., anhedonia) versus parent-child relationship factors (e.g., closeness). This work examined concurrent and prospective associations between youth neurophysiological reward responsiveness and parental anhedonia, parent-adolescent discord, and parent-adolescent closeness. Participants included 93 youth assigned female at birth (ages 13–15) and their mothers (*n* = 62 with a depression history). Youth reward responsiveness was assessed at baseline and one-year follow-up using the reward positivity (RewP) ERP component. Parental anhedonia, parent-adolescent discord, and parent-adolescent closeness were measured at each timepoint using questionnaires. Regression analyses demonstrated positive concurrent associations between parent-adolescent closeness and youth RewP at both timepoints. RewP was not significantly related to parental anhedonia or parent-adolescent discord, and no prospective cross-lagged effects were observed. Among adolescents at varying depression risk, youth with greater closeness with their mothers consistently demonstrated enhanced reward responsiveness, even after accounting for adolescent depressive symptoms and maternal depression history. Findings suggest that positive, but not negative, aspects of parent-child relationships are related to adolescent responsiveness to reward.

## Highlights

• Blunted neurophysiological reward response is a risk factor for depression in adolescence.
• Parental anhedonia, and parent-adolescent discord and cloessness may contribute to risk for youth blunted reward response.
• This study shows that daughters who share more closeness with their mother show enhanced neurophysiological reward response.
• Parent-adolescent closeness may be a protective factor for at-risk adolescent girls.

## 1 Introduction

Rates of depression rise markedly across the adolescent transition, particularly among daughters of mothers with a history of depression (Avenevoli et al., 2015, Hankin et al., 2015, Weissman et al., 2016). This elevated risk coincides with significant neurobiological changes, including the maturation of mesocorticolimbic reward systems, which underlie sensitivity to rewarding and motivationally salient experiences (Galvan et al., 2019, Larsen et al., 2020, Larsen and Luna, 2015). Disruptions in the development of these systems has been linked to individual differences in neurophysiological reward processing, a mechanism implicated in depression vulnerability (Kujawa, 2024, Kujawa and Burkhouse, 2017). Depression is characterized by decreased neural and behavioral reactivity to rewarding stimuli (Admon and Pizzagalli, 2015), and previous research indicates that adolescent daughters of depressed mothers demonstrated blunted neural responsiveness to reward compared with their typically developing, lower-risk peers (Burkhouse and Kujawa, 2023). Importantly, this work demonstrates that individual differences in neurophysiological responsiveness to reward—similar to those observed in major depressive disorder (MDD)—are present in high-risk girls prior to the onset of a first depressive episode, indicating that alterations in reward processing may function as a trait vulnerability factor predisposing these youth to risk for psychopathology.

Research using electroencephalography (EEG) demonstrates that neurophysiological indices of reward responsiveness can be reliably measured during adolescence using event related potentials (ERPs; Burkhouse and Kujawa, 2023; Kujawa, 2024; Luking et al., 2017). Specifically, the reward positivity (RewP) component, observed as a positive deflection at frontocentral electrode sites approximately 250–350ms following reward-related feedback, is a temporally-sensitive indicator of neurophysiological sensitivity to reward (Proudfit, 2015). The RewP is closely linked to activity in mesocorticolimbic reward circuity, including the ventral striatum, caudate, and medial prefrontal cortex (Carlson et al., 2011, Foti et al., 2015), and can be reliably elicited using laboratory-based monetary reward tasks (Levinson et al., 2017). The RewP may be disrupted in adolescents with or at high-risk for depression, reflecting altered reward system functioning (Kujawa, 2024). Consistent with this, the RewP has demonstrated robust associations with depression risk in childhood and adolescence (Keren et al., 2018, Kujawa, 2024).

Never-depressed offspring of depressed parents have been found to demonstrate blunted RewP (Burkhouse and Kujawa, 2023), yet specific mechanisms linking parental depression history with alterations in youth neurophysiological reward processing are not well understood. This gap is consequential, as the identification of specific factors within the parent-adolescent relationship that might relate to concurrent and prospective risk for neurophysiological reward dysfunction is needed to circumvent risk and bolster resilience in this high-risk population. Such factors may include alterations in parents’ own reward processing, as reflected in the presence of anhedonic symptoms of depression which are linked to individual differences in neural response to reward (Pizzagalli, 2014), as well as individual differences in parenting and the parent-adolescent relationship, as reflected in levels of parent-adolescent discord and closeness (Dix and Meunier, 2009); however, the role of these parent-adolescent risk factors in shaping concurrent and prospective alterations in adolescent RewP have not been rigorously evaluated. Thus, to advance knowledge of risk processes contributing to neurophysiological reward dysfunction in high-risk adolescent girls, the present study evaluated concurrent and prospective associations between maternal anhedonia, parent-adolescent discord, and parent-adolescent closeness and adolescent neurophysiological reward responsiveness, as assessed using the RewP, at two timepoints across one year.

### 1.1 Maternal anhedonia as a risk factor for offspring reward dysfunction

Anhedonia, or the pervasive loss of interest and/or pleasure, is a criterial symptom of depression that is related to alterations in behavioral and neural processes implicated in reward, including motivation, reward-based decision making, and effective reinforcement learning (Pizzagalli, 2014). Neuroimaging research finds that anhedonia associates with reduced activation in mesocorticolimbic reward circuitry, including the ventral and dorsal striatum and perigenual anterior cingulate cortex (pgACC; Pizzagalli, 2022). Thus, maternal anhedonia likely reflects aberrations in mothers’ own neural reward processing, which may disrupt normative developmental trajectories of reward processing in offspring, particularly during adolescence, a period when reward circuitry is undergoing significant maturation (Galvan et al., 2019, Larsen et al., 2020, Larsen and Luna, 2015). These disruptions may predispose offspring of anhedonic mothers to inherited or environmentally-mediated differences in neurophysiological reactivity to rewards.

Although a wealth of research has evaluated neural reward processing in offspring of depressed mothers, little work has homed in on unique effects of maternal anhedonia on adolescent reward dysfunction. One prior cross-sectional study conducted among psychiatrically healthy parent-adolescent dyads using functional magnetic resonance imaging (fMRI) found that maternal anhedonic symptoms were associated with reduced activation in the dorsolateral striatum and insula in response to monetary reward among early- to mid- adolescent daughters (Luking et al., 2019). These findings suggest that maternal anhedonia may influence activation in offspring mesocorticolimbic reward regions. Similarly, behavioral research indicates that parental anhedonia predicts relatively unique variance in adolescent risk for depression, controlling for other symptoms of parental depression (Griffith et al., 2024). Research is needed to extend these findings and evaluate concurrent and prospective associations between maternal anhedonia and youth neurophysiological reward response in daughters of mothers with a history of depression.

### 1.2 Alterations in the parent-adolescent relationship as a risk factor for offspring reward dysfunction

Alterations in neurophysiological reward response among daughters of depressed mothers are also likely to be influenced by more proximal behavioral experiences within the parent-adolescent relationship. Adolescence is a period of normative change in the parent-adolescent relationship characterized by increases in parent-adolescent discord and decreases in parent-adolescent closeness (Steinberg and Morris, 2001). At the same time, not all parent-adolescent dyads experience these changes equally, and individual differences in parent-adolescent discord and closeness may contribute to individual differences in biological risk processes (Hollenstein and Lougheed, 2013), including concurrent and prospective risk for adolescent reward-related dysfunction. Indeed, research in animal models supports associations between maternal caregiving and the development of neural and behavioral reward functioning among offspring (Duque-Quintero et al., 2022, Glaser, 2000). Individual differences in parent-adolescent discord and closeness may be especially salient for developmental outcomes among children of mothers with a history of depression, given robust associations between maternal depression history and negative alterations in parenting (Lovejoy et al., 2000).

Preliminary research provides support for associations between parent-adolescent relationship quality and youth neurophysiological reward response, as measured using the RewP. Among preschool-aged children, parental encouragement has been found to relate to an enhanced RewP (Kawamoto and Hiraki, 2019). Further, prospective work finds that low levels of positive parenting in early childhood predict blunted RewP in mid- to late- childhood, particularly among children of depressed caregivers (Kujawa et al., 2015). Interestingly, relatively less work has found support for associations between *negative* parent-adolescent interactions and alterations in youth neurophysiological reward response (see Kujawa et al., 2020), although it is notable that little research has been conducted among adolescents, who may be particularly sensitive to parent-child conflict relative to younger children (Weymouth et al., 2016).

### 1.3 Accounting for bidirectionality

The existing, albeit small, body of work evaluating prospective associations between parent-adolescent factors and youth RewP has predominantly focused on unidirectional effects of parent-adolescent factors on youth neurophysiological reward response over time. This research has yielded foundational insights into the role of parent-related factors in shaping youth reward-related neurophysiology. Yet, parent-adolescent relationships are bidirectional in nature (Pardini, 2008, Steinberg, 2001), and the relative contributions of youth reward-related neurophysiology to prospective change within the parent-adolescent relationship are not well understood. It is possible, for example, that blunted adolescent neurophysiological reward response contributes to individual differences in adolescent behavior (e.g., increased withdrawal) that exacerbate maternal depression and increase parent-adolescent discord over time. Reduced neurophysiological reward response may also interfere with youths’ ability to benefit from positive parent-adolescent interactions, impairing parent-adolescent closeness. To date, however, such prospective, bidirectional pathways have not been empirically tested. Clarifying potential bidirectional effect is important to identifying parent-adolescent processes contributing to “downward spirals” in youth socioemotional wellbeing.

### 1.4 The present study

To address gaps in the existing literature, the present study evaluated concurrent and prospective associations between youth neurophysiological reward response, as measured using the RewP, and maternal anhedonia, parent-adolescent discord, and parent-adolescent closeness in a risk-enhanced sample based on maternal history of depression. To assess potential bidirectional effects, youth RewP and parent-adolescent factors were assessed using an identical series of procedures at baseline and one-year later and were modeled using a cross-lagged panel model (CLPM) approach. CLPMs allow for the examination of prospective, bidirectional effects, accounting for the presence of autoregressive stability of constructs over time, as well as contemporaneous associations between predictors and outcomes at each timepoint.

Based on previous theory and research (e.g., Burkhouse and Kujawa, 2023; Dix and Meunier, 2009; Kujawa et al., 2020), we hypothesized that adolescent RewP would demonstrate negative concurrent and prospective associations with maternal anhedonia. We also hypothesized that adolescent RewP would demonstrate positive concurrent and prospective associations with parent-adolescent closeness. Given that previous research has not consistently supported associations between RewP and negative aspects of parent-child relationships (see Burkhouse and Kujawa, 2023), we made no a priori hypotheses regarding associations between adolescent RewP and parent-adolescent discord. We similarly made no a priori hypotheses regarding prospective effects of adolescent RewP on parent-adolescent outcomes.

## 2 Methods

### 2.1 Participants

Participants included 93 adolescents assigned female at birth and their biological mothers recruited for a longitudinal study examining risk factors in the development of depression. At baseline, adolescents were between the ages of 13 and 15 (M=13.80). Girls were not eligible for the study if they had a current or past DSM-5 diagnosis of any depressive disorder, a lifetime history of taking antidepressants (e.g. SSRIs), a lifetime presence of a DSM-5 psychotic, bipolar, or autism spectrum disorder, presence of EEG contraindications (e.g. personal lifetime history of seizures or family history of hereditary epilepsy), being pre-pubertal (< 3 Tanner Stage) as assessed by the Pubertal Development Scale (PDS; Petersen et al., 1988), a lifetime presence of a neurological or serious medical condition, presence of head injury or congenital neurological anomalies, an IQ < 70 (assessed using the Wechsler Abbreviated Scale of Intelligence (WASI)), uncorrected visual disturbance, and being acutely suicidal or at risk for harm to self and others. The sample was enriched for prospective risk for depression, as 66.7 % (*n* = 62) of adolescents had a mother with a history of major depression during their lifetime. Total family income (median=$95,000–100,000) was reported by mothers on a scale of 1–24 increments of $5,000 (1 = 0–5,000 to 24 = 115,000 +). Regarding adolescents’ race, 2 % self-identified as Asian American, 24 % as Black/African American, 9 % as Multiracial, 1 % as Native American, and 65 % as White. Regarding adolescents’ ethnicity, 2 % self-identified as Hispanic/Latine. No significant group differences (maternal depression: yes, no) were reported in age, pubertal stage, race/ethnicity, or socioeconomic status (lowest *p* = .07; see Table 1). Further details concerning recruitment and sample features can be found in Supplemental Materials (see Table S1).

**Table 1:** Participant demographics and descriptive characteristics of key study variables by group.

	Adolescents of Never Depressed Mothers (*n* = 31)	Adolescents of Depressed Mothers (*n* = 62)
Age	13.97 (.84)	13.71 (.80)
ALANA Identity	39 %	34 %
Family Income	$115000 +	$90,000–95000
T1 Maternal Anhedonia	52.24 (10.32)	62.25 (10.22)
T1 Parent-Adolescent Discord	2.73 (.56)	2.38 (.62)
T1 Parent-Adolescent Closeness	3.58 (.77)	3.56 (.62)
T1Adolescent Depression	7.10 (7.90)	9.33 (6.80)
T2 Maternal Anhedonia	48.80 (8.56)	60.02 (13.98)
T2 Parent-Adolescent Discord	2.37 (.59)	2.40 (.55)
T2 Parent-Adolescent Closeness	3.53 (.84)	3.40 (.66)
T2 Adolescent Depression	10.45 (10.78)	8.43 (7.61)

*Note.* Independent samples t-tests indicate that maternal anhedonia was higher among depressed relative to never-depressed mothers at both T1 and T2. No other variables were found to significantly differ between groups (lowest *p* = .07). ALANA = African/Black, Latino/a, Asian or Native American. All RewP and parent-adolescent variables were distributed relatively normally at each timepoint (skewness<|.79|, kurtosis<|.84|). Adolescent depression scores demonstrated mild positive skew (skewness=1.18 and 1.78, kurtosis=1.60 and 5.23 at T1 and T2, respectively).

### 2.2 Procedure

All study procedures were approved by the University of Pittsburgh Institutional Review Board. Mothers completed a phone screen to determine history of clinical depression for themselves and their adolescent, as well as other eligibility criteria. If both mother and adolescent were eligible, then they were invited to come to the University of Pittsburgh to complete a baseline appointment during which parental consent and adolescent assent were obtained. This initial baseline visit also included participating in clinical interviews, filling out questionnaires, and completing computerized tasks while wearing an EEG cap (adolescents only). This baseline appointment took approximately 6–8 h to complete. Participants were invited back to the lab approximately 12 months later to complete a follow-up assessment (T2) including questionnaires and adolescent EEG.^1^

### 2.3 Measures

#### 2.3.1 Maternal history of MDD

The Structured Clinical Interview for DSM-5 Disorders (SCID; First et al., 2015) was used to assess for mothers’ lifetime histories of depressive disorders.

#### 2.3.2 Maternal anhedonia

Mother’s self-reported current symptoms of anhedonia were assessed at baseline and 12-month follow-up using the anhedonic depression subscale of the Mood and Anxiety Symptom Questionnaire (MASQ; Watson and Clark, 1991). The Anhedonic Depression subscale of the MASQ includes 22 items assessing maternal experiences of anhedonia in the past week. For each item, participants were asked to rate the extent to which they experienced a given symptom on a scale from 1 (*Not at all*) to 5 (*Extremely)*. Internal consistency was good at both timepoints (omegas>.90).^2^

#### 2.3.3 Parent-adolescent discord

Parent-adolescent discord was assessed at baseline and 12-month follow-up using the discord subscale of the Network of Relationships Inventory – Relationship Qualities Version (NRI-RQV; Buhrmester and Furman, 2008). The Discord subscale of the NRI-RQV includes 15 items assessing negative features of parent-adolescent relationships including conflict, criticism, pressure, exclusion, and dominance. For each item, adolescents were prompted to rate the extent to which they experience specific features in their relationship with their mother from 1 (*Never or hardly at all*) to 5 (*Always or extremely much).* Internal consistency was good (omegas>.89).

#### 2.3.4 Parent-Adolescent closeness

Parent-adolescent closeness was assessed at baseline and 12-month follow-up using the closeness subscale of the Network of Relationships Inventory – Relationship Qualities Version (NRI-RQV; Buhrmester and Furman, 2008). The Closeness subscale of the NRI-RQV includes 15 items assessing positive features of parent-adolescent relationships including companionship, intimate disclosure, emotional support, approval, and satisfaction. For each item, adolescents were promoted to rate the extent to which they experience specific features in their relationship with their mother from 1 (*Never or hardly at all*) to 5 (*Always or extremely much*). Internal consistency was good (omegas>.93).

#### 2.3.5 Adolescent depression

Adolescent depression was assessed via self-report at baseline and 12-month follow-up using the Mood and Feelings Questionnaire – Child Version (MFQ-C; Angold et al., 1995). The MFQ includes 33 items assessing a range of youth depressive symptoms over the past 2 weeks. For each item, adolescents were asked to rate the extent to which a statement was true for them from 0 (*Not true*) to 2 (*True*). Internal consistency was good at both timepoints (omegas>.89).

#### 2.3.6 Reward positivity (RewP)

To assess reward responsivity, adolescents completed a “Doors Task”, which is a simple guessing game frequently used in research on reward processing (Levinson et al., 2017, Proudfit, 2015). At the start of each trial, participants are shown two doors and instructed to guess which door conceals a monetary prize by pressing the left or right button on a game controller. They are told that each correct guess results in winning 50¢, indicated by a green up-arrow, while each incorrect guess results in losing 25¢, indicated by a red down-arrow. Feedback was displayed for 2000 ms, followed by the message “Click for the next round”, which remained until the participant initiated the next trial. The task presented 30 win and 36 loss trials in random order, with a break halfway in between.^3^ All participants were told they could win up to $5 before beginning the task, and all participants were informed that they had won $5 in real money at the conclusion of the task. This sum was provided via payment card, in addition to other study payments.

### 2.4 EEG data recording and processing

Continuous EEG was recorded during the Doors Task using a custom cap and the BioSemi ActiveTwo system. The signal was pre-amplified at the electrode with a gain of 16x. The EEG was digitized at 24-bit resolution with a sampling rate of 512 Hz using a low-pass fifth-order sinc filter with a half-power cutoff of 104 Hz. Recordings were taken from 34 scalp electrodes based on the 10/20 system.

**Fig. 1:**
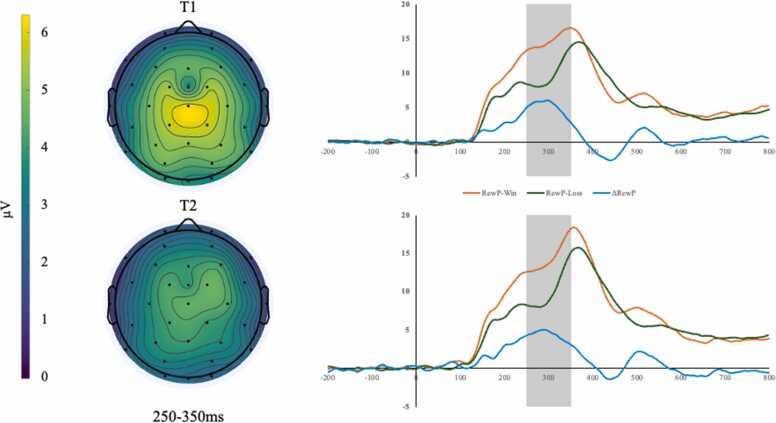
Scalp topography and grand-averaged RewP waveforms at site cz time-locked to receipt of the red or Green arrow. For the purposes of visualization, ΔRewP waveforms were calculated using a change score approach (RewP-Win – RewP-Loss), consistent with previous research (e.g., Tsypes et al., 2019; Mackin et al., 2023).

Off-line EEG analysis was performed using the MATLAB extension EEGLAB (Delorme and Makeig, 2004) and the EEGLAB plug-in ERPLAB (Lopez-Calderon and Luck, 2014). All data were band-pass filtered with cutoffs of 0.1 Hz and 30 Hz and re-referenced to the average of the left and right mastoid electrodes. EEG data were processed using both artifact rejection and correction. First, large and stereotypical ocular components were identified and removed using independent component analysis (ICA) scalp maps (Jung et al., 2001). Epochs were then extracted from raw EEG with the interval from −200ms to 0 ms serving as the baseline for each trial. Epochs with large artifacts (greater than 200 μV) were excluded from analysis. Consistent with prior research (Luking et al., 2017, Nelson et al., 2018, Pegg et al., 2020), the RewP for wins and losses was scored as the mean activity from 250–350 ms after stimulus onset at electrode site Cz. To be included in analyses, informed by prior psychometric research (Luking et al., 2017), participants had to retain at least 14 trials from each win and loss category. A criterion of 14 minimum trials has been fruitfully applied to understand neurophysiological correlates of risk in youth (Tsypes et al., 2019). Consistent with previous work (e.g., Duttweiler et al., 2024), RewP was represented in analyses as the standardized residual of RewP in response to wins controlling for RewP in response to loss. Grand-averaged RewP waveforms time-locked to receipt of the green or red arrow and the corresponding scalp topography are illustrated in Fig. 1.^4^ The even-odd split-half reliability of RewP responses to win and loss trials was adequate (Guttman split-half coefficients =.85–.94 and.67–.85, respectively).”

### 2.5 Data analytic plan

**Fig. 2:**
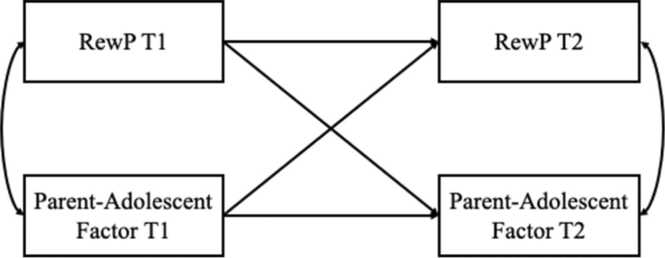
Sample structural model corresponding to analyses of prospective, bidirectional effects between RewP and parent-adolescent factors across one year.

Hypotheses were tested using a series of structural regressions implemented using the ‘lavaan’ library in R (R Core Team, 2022, Rosseel, 2012) using maximum likelihood robust (MLR) estimator to account for nonnormality and full information likelihood estimation (FIML) to account for missing data. First, to examine contemporaneous associations between RewP and parent-adolescent factors, we conducted a series of regressions in which RewP at a given time point was regressed on maternal anhedonia, parent-adolescent discord, and parent-adolescent closeness at the same time point. Predictors were examined in separate models in the interest of power, and 95 % confidence intervals were used, in addition to null hypothesis significance testing, in interpretations of effects. Preliminary analyses indicated that maternal MDD status did not moderate effects (all *p*s > .05); thus, maternal MDD (0 =lifetime absent, 1 =lifetime present) was included as a covariate in these analyses given evidence that maternal depression is related to both individual differences in youth RewP (Burkhouse and Kujawa, 2023), as well as individual differences in parenting relevant to parent-adolescent discord and closeness (Lovejoy et al., 2000). Maternal MDD was also oversampled in the current study. Adolescent contemporaneous depressive symptoms were also included as covariates in these analyses to ensure that observed effects were not artifacts of youths’ own depression. Finally, we covaried for age due to established associations between age and parent-adolescent closeness and discord (Steinberg and Morris, 2001), ensuring that any observed associations between parent-adolescent closeness and discord and adolescent RewP were not better accounted for variance shared with adolescent age. Next, to evaluate prospective, bidirectional associations between RewP and parent-adolescent factors, we conducted a series of CLPMs, accounting for autoregressive effects, as well as contemporaneous correlations between RewP and parent-adolescent factors at each time point (see Fig. 2). As in previous analyses, parent-adolescent predictors were modeled separately in the interest of power and interpretability.

## 3 Results

### 3.1 Preliminary analyses

Descriptive characteristics for primary variables of interest are reported in Table 1. Mothers with a history of depression reported significantly higher anhedonia at each time point relative to mothers with no lifetime history of depression (*p*s < .05). No other mean-level differences were observed between groups. Correlations between primary variables of interest are reported in Table 2. Parent-adolescent discord and closeness were negatively related at each timepoint.

**Table 2:** Correlations between primary variables of interest.

	1	2	3	4	5	6	7	8	9	10
1. T1 RewP residual	--									
2. T1 Maternal anhedonia	.07	--								
3. T1 Parent-adolescent discord	−.17	−.08	--							
4. T1 Parent-adolescent closeness	.28*	−.02	−.48*	--						
5. T1 Adolescent depression	−.08	.07	.35*	−.17	--					
6. T2 RewP residual	.36*	.15	−.07	.20	.18	--				
7. T2 Maternal anhedonia	.04	.66*	−.18	.05	.01	.25	--			
8. T2 Parent-adolescent discord	−.13	−.11	.66*	−.36*	.36*	−.17	−.05	--		
9. T2 Parent-adolescent closeness	.20	−.05	−.31*	.55*	−.38	.27	.04	−.52*	--	
10. T2 Adolescent depression	.18	.03	.15	−.01	.47*	.07	.07	.37*	−.17	--
11. T1 Adolescent age	−.08	.15	−.14	−.02	.06	.04	.33*	−.01	−.01	.09

*Note*. *p < .05

### 3.2 Contemporaneous associations between RewP and parent-adolescent factors

**Fig. 3:**
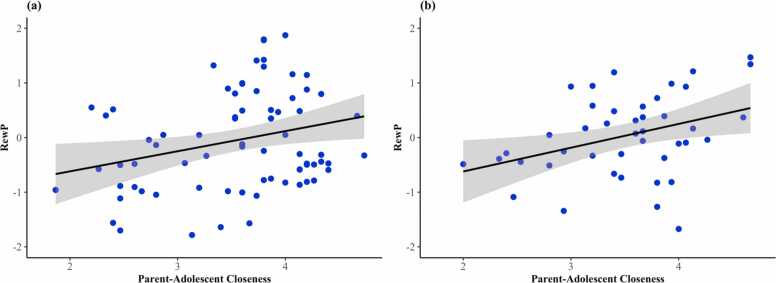
Scatterplots representing cross-sectional associations between parent-adolescent closeness and RewP at time 1 (a) and time 2 (b). shaded bars indicate the 95 % confidence interval of the estimate.

Results of regressions evaluating contemporaneous associations between variables at T1 and T2 are reported in Table 3, Table 4, respectively. Controlling for maternal MDD history, adolescent age, and adolescent symptoms of depression, parent-adolescent closeness demonstrated a significant positive contemporaneous association with RewP at T1 (β=.27, *p* = .003) and T2 (β=.30, *p* = .037). Bivariate associations between RewP and parent-adolescent closeness at each timepoint are depicted in Fig. 3. Parent-adolescent discord and maternal anhedonia were not associated with RewP at either time point (*p*s > .05).

**Table 3:** Results of regressions evaluating concurrent associations between RewP and parent-adolescent variables at time 1.

	β	*b*	SE(*b*)	95 % CI	*p*
**Model 1: RewP on Maternal Anhedonia**					
Maternal MDD	.06	.12	.25	[−.37,.61]	.630
Adolescent depression	−.10	−.01	.02	[−.04,.02]	.430
Adolescent age	−.04	−.04	.11	[−.26,.19]	.730
Maternal anhedonia	.07	.01	.01	[−.02,.03]	.615
**Model 2: RewP on Parent-Adolescent Discord**					
Maternal MDD	.10	.20	.21	[−.21,.62]	.340
Adolescent depression	−.02	−.003	.02	[−.04,.04]	.879
Adolescent age	−.06	−.07	.12	[−.30,.16]	.551
Parent-adolescent discord	−.18	−.29	.17	[−.62,.04]	.085
**Model 3: RewP on Parent-Adolescent Closeness**					
Maternal MDD	.09	.17	.21	[−.25,.58]	.429
Adolescent depression	−.04	−.01	.02	[−.04,.03]	.776
Adolescent age	−.03	−.03	.11	[−.24,.18]	.782
Parent-adolescent closeness	.27	.38	.13	[.13,.64]	.003

*Note.* Β=standardized effect size; *b*=unstandardized effect size; SE(*b*)=standard error of the unstandardized effect; 95 % CI= 95 % confidence interval for the unstandardized effect.

**Table 4:** Results of regressions evaluating concurrent associations between RewP and parent-adolescent variables at time 2.

	β	*b*	SE(*b*)	95 % CI	*p*
**Model 1: RewP on Maternal Anhedonia**					
Maternal MDD	.12	.24	.33	[−.40,.89]	.461
Adolescent depression	.13	.01	.02	[−.02,.05]	.373
Adolescent age	.02	.03	.24	[−.43.49]	.903
Maternal anhedonia	.20	.02	.01	[−.01,.04]	.218
**Model 2: RewP on Parent-Adolescent Discord**					
Maternal MDD	.16	.36	.35	[−.32, 1.04]	.295
Adolescent depression	.16	.02	.02	[−.01,.05]	.194
Adolescent age	.04	.05	.21	[−.36,.45]	.821
Parent-adolescent discord	−.20	−.37	.25	[−.87,.13]	.145
**Model 3: RewP on Parent-Adolescent Closeness**					
Maternal MDD	.19	.41	.34	[−.26, 1.07]	.233
Adolescent depression	.18	.02	.01	[−.01,.05]	.120
Adolescent age	.01	.01	.21	[−.40,.43]	.950
Parent-adolescent closeness	.30	.42	.20	[.03,.81]	.037

*Note.* Β=standardized effect size; *b*=unstandardized effect size; SE(*b*)=standard error of the unstandardized effect; 95 % CI= 95 % confidence interval for the unstandardized effect.

### 3.3 Prospective bidirectional associations between RewP and Parent-Adolescent factors

Results of CLPMs evaluating prospective, bidirectional associations are reported in Table 5. Significant autoregressive effects indicate that RewP demonstrated moderate rank order stability across one year. Moderately large autoregressive effects were also observed for maternal anhedonia, parent-adolescent discord, and parent-adolescent closeness (see Table 5). No cross-lagged effects between RewP and parent-adolescent factors were observed (all *p*s > .05).

**Table 5:** Results of cross-lagged analyses evaluating prospective bidirectional associations between RewP and parent-adolescent variables.

	β	*b*	SE(*b*)	95 % CI	*p*
**Model 1: RewP and Maternal Anhedonia**					
RewP T1 → RewP T2	.34	.33	.09	[.15,.52]	< .001
Maternal anhedonia T1 → Maternal anhedonia T2	.65	.72	.12	[.48.96]	< .001
Maternal anhedonia T1 → RewP T2	.10	.01	.01	[−.01,.03]	.441
RewP T1 → Maternal Anhedonia T2	−.03	−.33	1.37	[−3.02, 2.36]	.811
**Model 2: RewP and Parent-Adolescent Discord**					
RewP T1 → RewP T2	.34	.33	.10	[.14.53]	.001
Parent-adolescent discord T1 → Parent-adolescent discord T2	.66	.62	.09	[.45,.80]	< .001
Parent-adolescent discord T1 → RewP T2	−.01	−.02	.22	[−.44,.40]	.925
RewP T1 → Parent-adolescent discord T2	−.05	−.03	.06	[−.15,.10]	.678
**Model 3: RewP and Parent-Adolescent Closeness**					
RewP T1 → RewP T2	.31	.31	.10	[.11,.52]	.003
Parent-adolescent closeness T1 → Parent adolescent closeness T2	.52	.56	.14	[.28,.83]	< .001
Parent-adolescent closeness T1 → RewP T2	.10	.15	.23	[−.30,.59]	.513
RewP T1 → Parent-adolescent closeness T2	.04	.03	.07	[−.11,.16]	.686

*Note.* Β=standardized effect size; *b*=unstandardized effect size; SE(*b*)=standard error of the unstandardized effect; 95 % CI= 95 % confidence interval for the unstandardized effect.

## 4 Discussion

Neurophysiological responsiveness to reward is concurrently and prospectively associated with risk for depression (Keren et al., 2018; Kujawa et al., 2024) and has been found to be impaired in daughters of depressed mothers (Burkhouse and Kujawa, 2023). The parent-adolescent relationship is an important context in which youth affective development occurs (Griffith et al., 2021, Morris et al., 2017), yet specific features of the parent-adolescent relationship contributing to adolescent neurophysiological reward response are not well understood. Thus, this study evaluated concurrent and prospective relations between youth neurophysiological reward response, as measured using the RewP, and several salient features of the parent-adolescent relationship including parental anhedonia, parent-adolescent discord, and parent-adolescent closeness in a risk-enhanced sample based on maternal depression history. Findings indicate that regardless of maternal depression history and youths’ own symptoms of depression, adolescents who enjoy close, confiding relationships with their mothers demonstrate enhanced responsiveness to monetary rewards. Interestingly, no evidence for prospective or bidirectional effects between youth RewP and parent-adolescent factors were observed. Findings have implications for theories of intergenerational transmission of reward dysfunction in parent-adolescent dyads.

Adolescent RewP was found to demonstrate contemporaneous associations with parent-adolescent closeness at both baseline and 12-month follow-up. Importantly, associations between parent-adolescent closeness and adolescent RewP were maintained after controlling for maternal depression history and youth co-occurring depressive symptoms. This suggests that positive experiences within the parent-adolescent relationship may play a unique role in bolstering youth neurophysiological reward function during adolescence. This finding is consistent with previous research indicating relatively specific associations between positive parenting experiences (compared with negative parenting experiences) in early childhood and youth RewP (e.g., Kujawa et al., 2015; see Kujawa et al., 2020). It is also consistent with research in animal models demonstrating associations between early-life impairments in the quality and consistency of caregiving and later-life responsiveness to rewards (Duque-Quintero et al., 2022). The present findings build on this previous work by illustrating that parent-adolescent closeness is relatively uniquely important to youth neurophysiological reward response beyond early childhood into mid- to late- adolescence, suggesting that positive experiences within the parent-adolescent relationship may promote wellbeing and reduce affective risk among youth at varying risk for depression. It may be, for example, that experiencing emotional support and approval from one’s parent enhances adolescents’ ability to tap into rewarding qualities of other positive experiences outside of the family context, scaffolding youths’ ability to actively and effectively engage with positive environmental opportunities.

Interestingly, parental anhedonia and parent-discord were not related to youth RewP in the present work. These findings diverge from previous work using fMRI demonstrating associations between maternal anhedonia and adolescent neural reward response (Luking et al., 2019). Differences in patterns of findings may be related to differences in methodologies, as ERP provides precise, temporally-sensitive insights into electrophysiological responses to reward, whereas fMRI provides direct, spatially-specific insights into activation in underlying mesocorticolimbic regions. It is also possible that the associations between adolescent RewP and maternal anhedonia may only emerge among dyads with a parental history of threshold anhedonia, which was not well-captured using our dimensional measure. Of note, however, the present findings are consistent with previous work indicating that positive (but not negative) aspects of parent-adolescent relationships are reciprocally related to youth positive affect across the adolescent transition (Griffith et al., 2021), as well as research in younger children indicating that positive parenting (compared with negative parenting) may be a relatively specific predictor of youth RewP (see Kujawa et al., 2020). That maternal anhedonia was not found to relate to adolescent RewP might suggest that relatively proximal behavioral experiences within the parent-adolescent relationship may be more relevant to understanding youth contemporaneous reward function relative to more distal, underlying parental pathologies.

Contrary to expectations, no prospective effects of parent-adolescent factors on youth RewP were observed, nor were bidirectional effects from youth RewP to parent-adolescent factors supported. Of note, RewP demonstrated moderate levels of stability across the one-year follow-up period, and it may be that patterns of neurophysiological reward response have already crystallized by mid-adolescence. This is consistent with conceptualizations of the RewP as a trait vulnerability factor for psychopathology (Kujawa, 2024). Similarly, parental anhedonia, as well as parent-adolescent discord and closeness were highly stable over time, limiting variance to detect prospective bidirectional effects and indicating a restricted range of change in these variables over time. Conversely, it is possible that the timescale of the present work was not optimally calibrated to detect prospective effects. That is, it may be that parent-adolescent factors and youth RewP demonstrate bidirectional influence over more a protracted period of development (i.e., > 1 year). Future work is needed to explore this possibility.

Interestingly, no main effects of group status (i.e., high- versus low- risk) on RewP were observed. This diverges from previous research indicating blunted RewPs in daughters of depressed mothers (see Burkhouse and Kujawa, 2023). Of note, however, the sample size of low-risk girls in the present work was limited, and it may be that we were underpowered to detected group-level effects.

The present study benefits from several strengths including its repeated-measures design, risk-enhanced sample, and inclusion of multiple informants. Together, these strengths allowed the present study to rigorously evaluate concurrent and bidirectional associations between parent-adolescent factors and adolescent RewP among vulnerable youth, advancing knowledge of risk processes across the adolescent transition. The present findings should also be interpreted in light of several limitations, which reflect important areas for future research. First, the present study evaluated maternal anhedonia, measured dimensionally, as a proxy measure for parental reward dysfunction. Future work is needed directly evaluating parental neurophysiological response to reward (e.g., using ERPs) to more rigorously evaluate the role of impairments in parental reward response in shaping developmental outcomes during adolescence. Future research may also benefit from using interview measures of parental anhedonia, such as the SCID, to examine differences in neurophysiological reward responsiveness among daughters of mothers with and without clinically significant levels of anhedonia. Second, although the present sample size matches or exceeds that of other risk-enhanced samples of similarly aged youth (e.g., Duttweiler et al., 2024), power may have nevertheless been limited to detect potential interactive effects of maternal depression history on associations of interest. The median income of participants in the present sample also exceeds community norms, and the extent to which findings generalize to lower resourced families is unclear. Research is needed in larger, more socially diverse samples to evaluate the extent to which parent-adolescent closeness or discord may be more or less relevant to youth RewP amongst youth at high- or lower- familial risk for depression. Additionally, the present study excluded youth with a history of a depressive disorder. This sampling feature, while important for advancing knowledge of risk processes preceding disorder onset in high-risk girls, may nevertheless have resulted in an oversampling of high-resilience youth, potentially limiting generalizability of findings. Finally, the present study employed broad measures of parent-adolescent closeness and discord. This design choice provides important foundational insights into features of the parent-adolescent relationship most relevant to youth reward response; however, research is needed using more fine-grained measures to clarify specific behavioral processes (e.g., parental praise, co-regulation of positive emotions) contributing to RewP during adolescence.

Using a prospective, repeated-measures study design, the present work demonstrates that parent-adolescent closeness demonstrates relatively unique contemporaneous associations with youth neurophysiological reward response during adolescence. Findings suggest that positive aspects of parent-adolescent relationships continue to be relevant to healthy adolescent development through mid- to late- adolescence and indicate that attending to positive parent-adolescent relationships may be especially important to understanding neurophysiological mechanisms of risk among youth at varying risk for depression.

## CRediT authorship contribution statement

**Julianne M. Griffith:** Writing – review & editing, Writing – original draft, Visualization, Validation, Software, Methodology, Formal analysis, Conceptualization. **Anna Wears:** Writing – review & editing, Resources, Project administration, Investigation, Data curation, Conceptualization. **Nastasia O. McDonald:** Writing – review & editing, Resources, Project administration, Investigation, Data curation, Conceptualization. **Jennifer S. Silk:** Writing – review & editing, Supervision, Methodology, Funding acquisition, Conceptualization. **Rebecca B. Price:** Writing – review & editing, Supervision, Methodology, Funding acquisition, Conceptualization. **Mary L. Woody:** Writing – review & editing, Writing – original draft, Visualization, Validation, Supervision, Software, Resources, Project administration, Methodology, Investigation, Funding acquisition, Formal analysis, Data curation, Conceptualization.

## Funding

This work was supported by the 10.13039/100000002National Institutes of Health (K23 MH119225; PI: Mary L. Woody). Julianne M. Griffith is supported by T32 MH018269.

## Declaration of Competing Interest

The authors declare that they have no known competing financial interests or personal relationships that could have appeared to influence the work reported in this paper.

## Footnotes

Appendix A Supplementary data associated with this article can be found in the online version at doi:10.1016/j.dcn.2025.101579.

## Footnotes

1 Further details regarding participant retention are provided in Supplemental Materials.

2 Descriptive statistics corresponding to other subscales of the MASQ are reported in Supplemental Table S2 to provide further descriptive information regarding the study sample.

3 Due to a coding error, the version of the Doors task administered in the present study included six additional loss trials, resulting in a non-equivalent number of win and loss trials. Although unanticipated, we do not believe that the addition of these extra loss trials threatens the conceptual or methodological integrity of the task. We acknowledge this deviation, however, as a limitation of the present work.

4 Complementary analyses were conducted examining the RewP loss residual (i.e., the standardized residual of RewP response to loss controlling for RewP response to wins). Findings indicated no concurrent or prospective associations between RewP to loss and parent-adolescent factors. Complete results of these analyses are reported in Supplemental Tables S3–5.
