# Supplementary material for: Parent-adolescent closeness predicts neurophysiological reward responsiveness in adolescent girls at varying risk for depression
Source: Dev Cogn Neurosci. 2025 Jun 3;74:101579. doi: 10.1016/j.dcn.2025.101579 (PMC12173130; doi:10.1016/j.dcn.2025.101579)
Supplement: Supplementary file 1 — Supplementary material [file mmc1.docx]

**Supplemental Materials**

**Participant Recruitment**

Mothers and their daughters were recruited through flyer and internet advertisement as well as through the University of Pittsburgh research portal. Girls were not eligible for the study if they had a current or past DSM-5 diagnosis of any depressive disorder, a lifetime history of taking antidepressants (e.g. SSRIs), a lifetime presence of a DSM-5 psychotic, bipolar, or autism spectrum disorder, presence of EEG contraindications (e.g. personal lifetime history of seizures or family history of hereditary epilepsy), being pre-pubertal (< 3 Tanner Stage) as assessed by the Pubertal Development Scale (PDS; Peterson et al., 1988), a lifetime presence of a neurological or serious medical condition, presence of head injury or congenital neurological anomalies, an IQ <70 (assessed using the Wechsler Abbreviated Scale of Intelligence (WASI)), uncorrected visual disturbance, and being acutely suicidal or at risk for harm to self and others. Additionally, mothers were not eligible for the study if they had a current or past DSM-5 diagnosis of schizophrenia or bipolar disorder. Both mothers with and without a history of MDD were recruited for the study, but a mother was not eligible if her depressive episode(s) exclusively fell outside of her daughter’s lifetime.

**Participant Retention**

Data from 69 participants (74.2% retention; *n*=45 adolescents of depressed mothers) was available at T2. Participants who completed T2 did not differ from participants who did not complete T2 based on maternal depression history, maternal anhedonia, parent-adolescent closeness, parent-adolescent discord, or RewP at baseline. Participants who returned for T2 differed in age than those who did not (*t*(df)=2.35(91), *p*=.021), such that retained youth (M[SD]=13.68[.80]) were slightly younger at baseline than those who did not complete the study (M[SD]=14.13[.80]). Of note, the study was conducted during the COVID-19 pandemic, and families were offered the option to complete remote follow-ups in the interest of health and safety. We expect that these public health concerns may have introduced limitations to the present retention rates.

In terms of missing data, six adolescents declined to participate in the Doors task, or were unable to complete the Doors task due to time constraints or equipment failure at T1. Further, Doors Task data from two participants at T1 and one participant at T2 were excluded due to a high number of artifacts. Therefore, data from 86 adolescents (92.5%) were available for analysis at T1, and 58 adolescents (96.7%) at T2.^[[1]](#footnote-1)^ Of questionnaire data, 89 (95.7%) parents provided responses on the MASQ and 84 (90.3%) adolescents provided responses on the NRI-RQV at T1. Questionnaire data from 60 participants (87.0%) was available at T2.

**Interviewer Training and Supervision**

All clinical interviews were conducted by post-baccalaureate research staff trained and supervised by the principal investigator (MLW), a licensed clinical psychologist. Prior to participating in interviews, staff received extensive didactic instruction, reviewed video, and were required to observe an expert interviewer in a live interview setting. For training purposes, trainees were required to code recorded interviews and achieve reliability with established interviewers. After achieving reliability based on recorded interviews and observing at least five live interviews, interviewers received live supervision for their first 10 interviews with study participants to ensure adequate fidelity to study procedures and enhance reliability. MLW provided ongoing supervision for the duration of the study. A second interviewer independently coded a subset of 10 SCID interviews, and inter-rater reliability for MDD diagnoses was excellent (κs = 1.00).

Table S1

*Participant Diagnostic History by Group*

|  | Adolescents of Never Depressed Mothers (*n*=30) | Adolescents of Depressed Mothers (*n*=61) |
| --- | --- | --- |
| Adjustment Disorder – Current | 1 | 1 |
| Adjustment Disorder – Lifetime | 4 | 3 |
| Anxiety Disorder – Current | 16 | 39 |
| Anxiety Disorder – Lifetime | 18 | 42 |
| OCD – Current | 1 | 3 |
| OCD – Lifetime | 1 | 3 |
| PTSD - Current | 1 | 2 |
| PTSD - Lifetime | 2 | 3 |
| Eating Disorder – Current | 3 | 2 |
| Eating Disorder - Lifetime | 3 | 2 |
| ADHD – Current | 1 | 8 |
| ADHD - Lifetime | 1 | 8 |
| ODD – Current | 0 | 4 |
| ODD – Lifetime | 0 | 4 |

*Note.* Anxiety disorders included panic disorder, agoraphobia, separation anxiety disorder, specific phobia, social anxiety disorder, and generalized anxiety disorder. Among participants with current anxiety disorder diagnoses, 42.10% (*n*=24) were diagnosed with two or more discrete disorders; comorbidity rates among participants with a lifetime anxiety disorder diagnosis were 45.16% (*n*=28). Chi-square difference tests indicated no significant group differences in diagnoses across diagnostic categories (all *p*s>.05).

Table S2

*Means and standard deviations describing non-anhedonia subscales of the Mood and Anxiety Symptom Questionnaire (MASQ) by group*

|  | Never Depressed Mothers (*n*=31) | Depressed Mothers (*n*=62) |
| --- | --- | --- |
| T1 General Distress – Anxiety | 13.83 (2.78) | 17.78 (5.32) |
| T2 General Distress – Anxiety | 14.40 (2.89) | 17.18 (4.99) |
| T1 Anxious Arousal | 18.77 (2.40) | 21.42 (4.61) |
| T2 Anxious Arousal | 18.25 (1.62) | 20.83 (4.60) |
| T1 General Distress – Depression | 15.87 (3.61) | 22.44 (7.25) |
| T2 General Distress – Depression | 16.05 (2.87) | 21.63 (9.06) |
| T1 General Distress – Mixed | 29.70 (5.66) | 40.10 (11.81) |
| T2 General Distress - Mixed | 30.35 (5.24) | 38.80 (12.57) |

*Note.* Independent samples t-tests indicate that all symptom domains were significantly higher among mothers with a history of depression relative to never-depressed mothers at both timepoints (all *p*s<.01).

Table S3

*Results of regressions evaluating concurrent associations between RewP to loss and parent-adolescent variables at Time 1*

|  | β | *b* | SE(*b*) | 95% CI | *p* |
| --- | --- | --- | --- | --- | --- |
| **Model 1: RewP - Loss on Maternal Anhedonia** | | | | | |
| Maternal MDD | .17 | .35 | .25 | [-.13, .84] | .155 |
| Adolescent depression | -.05 | -.01 | .02 | [-.04, .03] | .705 |
| Adolescent age | .04 | .04 | .12 | [-.19, .28] | .716 |
| Maternal anhedonia | .11 | .01 | .01 | [-.01, .03] | .421 |
| **Model 2: RewP – Loss on Parent-Adolescent Discord** | | | | | |
| Maternal MDD | .24 | .51 | .22 | [.08, .94] | .022 |
| Adolescent depression | .05 | .01 | .02 | [-.03, .04] | .698 |
| Adolescent age | .09 | .11 | .12 | [-.13, .35] | .364 |
| Parent-adolescent discord | -.08 | -.14 | .25 | [-.63, .36] | .591 |
| **Model 3: RewP - Loss on Parent-Adolescent Closeness** | | | | | |
| Maternal MDD | .24 | .50 | .22 | [.06, .93] | .025 |
| Adolescent depression | .03 | .04 | .02 | [-.03, .04] | .831 |
| Adolescent age | .10 | .13 | .12 | [-.10, .36] | .282 |
| Parent-adolescent closeness | .04 | .06 | .16 | [-.25, .36] | .714 |

*Note.* Β=standardized effect size; *b*=unstandardized effect size; SE(*b*)=standard error of the unstandardized effect; 95% CI=95% confidence interval for the unstandardized effect.

Table S4

*Results of regressions evaluating concurrent associations between RewP to loss and parent-adolescent variables at Time 2*

|  | β | *b* | SE(*b*) | 95% CI | *p* |
| --- | --- | --- | --- | --- | --- |
| **Model 1: RewP - Loss on Maternal Anhedonia** | | | | | |
| Maternal MDD | .12 | .25 | .29 | [-.32, .81] | .390 |
| Adolescent depression | -.01 | -.001 | .02 | [-.04, .03] | .969 |
| Adolescent age | .12 | .15 | .21 | [-.25, .56] | .461 |
| Maternal anhedonia | .38 | .02 | .01 | [-.003, .04] | .081 |
| **Model 2: RewP - Loss on Parent-Adolescent Discord** | | | | | |
| Maternal MDD | .16 | .32 | .29 | [-.24, .88] | .260 |
| Adolescent depression | .08 | .01 | .02 | [-.03, .04] | .599 |
| Adolescent age | .12 | .14 | .18 | [-.21, .49] | .420 |
| Parent-adolescent discord | -.12 | -.21 | .28 | [-.77, .34] | .454 |
| **Model 3: RewP - Loss on Parent-Adolescent Closeness** | | | | | |
| Maternal MDD | .17 | .35 | .30 | [-.25, .94] | .252 |
| Adolescent depression | .06 | .01 | .02 | [-.02, .04] | .648 |
| Adolescent age | .09 | .11 | .18 | [-.24, .45] | .541 |
| Parent-adolescent closeness | .25 | .33 | .25 | [-.16, .83] | .183 |

*Note.* Β=standardized effect size; *b*=unstandardized effect size; SE(*b*)=standard error of the unstandardized effect; 95% CI=95% confidence interval for the unstandardized effect.

Table S5

*Results of cross-lagged analyses evaluating prospective bidirectional associations between RewP to loss and parent-adolescent variables*

|  | β | *b* | SE(*b*) | 95% CI | *p* |
| --- | --- | --- | --- | --- | --- |
| **Model 1: RewP - Loss and Maternal Anhedonia** | | | | | |
| RewP T1 → RewP T2 | .68 | .67 | .07 | [.52, .82] | <.001 |
| Maternal anhedonia T1 → Maternal anhedonia T2 | .64 | .71 | .12 | [.48, .95] | <.001 |
| Maternal anhedonia T1 → RewP T2 | -.05 | -.004 | .01 | [-.02, .02] | .662 |
| RewP T1 → Maternal Anhedonia T2 | .05 | .62 | 1.04 | [-1.42, 2.66] | .549 |
| **Model 2: RewP - Loss and Parent-Adolescent Discord** | | | | | |
| RewP T1 → RewP T2 | .67 | .67 | .07 | [.53, .81] | <.001 |
| Parent-adolescent discord T1 → Parent-adolescent discord T2 | .67 | .63 | .10 | [.44, .82] | <.001 |
| Parent-adolescent discord T1 → RewP T2 | -.08 | -.13 | .16 | [-.43, .18] | .420 |
| RewP T1 → Parent-adolescent discord T2 | -.05 | -.03 | .06 | [-.15, .10] | .677 |
| **Model 3: RewP - Loss and Parent-Adolescent Closeness** | | | | | |
| RewP T1 → RewP T2 | .68 | .68 | .07 | [.54, .83] | <.001 |
| Parent-adolescent closeness T1 → Parent adolescent closeness T2 | .54 | .59 | .14 | [.31, .87] | <.001 |
| Parent-adolescent closeness T1 → RewP T2 | .05 | .07 | .15 | [-.23, .37] | .647 |
| RewP T1 → Parent-adolescent closeness T2 | .14 | .10 | .06 | [-.02, .22] | .115 |

*Note.* Β=standardized effect size; *b*=unstandardized effect size; SE(*b*)=standard error of the unstandardized effect; 95% CI=95% confidence interval for the unstandardized effect.

1. One outlier was detected in T1 RewP data. Thus, additional sensitivity analyses were conducted excluding this participant. Results did not change when this value was excluded from the data. [↑](#footnote-ref-1)
